# Oocyte Competence Biomarkers Associated With Oocyte Maturation: A Review

**DOI:** 10.3389/fcell.2021.710292

**Published:** 2021-08-30

**Authors:** Batara Sirait, Budi Wiweko, Ahmad Aulia Jusuf, Dein Iftitah, R. Muharam

**Affiliations:** ^1^Department of Obstetrics and Gynecology, Faculty of Medicine, Universitas Kristen Indonesia, Jakarta, Indonesia; ^2^Morula IVF Jakarta Clinic, Jakarta, Indonesia; ^3^Division of Reproductive Endocrinology and Infertility, Department of Obstetrics and Gynecology, Faculty of Medicine, Universitas Indonesia, Jakarta, Indonesia; ^4^Yasmin IVF Clinic, Dr. Cipto Mangunkusumo General Hospital, Jakarta, Indonesia; ^5^Human Reproductive, Infertility, and Family Planning Research Cluster, Indonesia Medical Education and Research Institute (IMERI), Faculty of Medicine, Universitas Indonesia, Jakarta, Indonesia; ^6^Department of Histology, Faculty of Medicine, Universitas Indonesia, Jakarta, Indonesia

**Keywords:** oocyte competence, oocyte maturation, cumulus-oocyte complex (COC), biomarker, *in-vitro* fertilization

## Abstract

Oocyte developmental competence is one of the determining factors that influence the outcomes of an IVF cycle regarding the ability of a female gamete to reach maturation, be fertilized, and uphold an embryonic development up until the blastocyst stage. The current approach of assessing the competency of an oocyte is confined to an ambiguous and subjective oocyte morphological evaluation. Over the years, a myriad of biomarkers in the cumulus-oocyte-complex has been identified that could potentially function as molecular predictors for IVF program prognosis. This review aims to describe the predictive significance of several cumulus-oocyte complex (COC) biomarkers in evaluating oocyte developmental competence. A total of eight acclaimed cumulus biomarkers are examined in the study. RT-PCR and microarray analysis were extensively used to assess the significance of these biomarkers in foreseeing oocyte developmental competence. Notably, these biomarkers regulate vital processes associated with oocyte maturation and were found to be differentially expressed in COC encapsulating oocytes of different maturity. The biomarkers were reviewed according to the respective oocyte maturation events namely: nuclear maturation, apoptosis, and extracellular matrix remodeling, and steroid metabolism. Although substantial *in vitro* evidence was presented to justify the potential use of cumulus biomarkers in predicting oocyte competency and IVF outcomes, the feasibility of assessing these biomarkers as an add-on prognostic procedure in IVF is still restricted due to study challenges.

## Introduction

Controlled ovarian stimulation is a critical step of a successful IVF cycle conducted to stimulate the development of multiple follicles in the ovaries, thereby increasing the chances of harvesting viable eggs that could potentially become viable blastocysts. Depending on the patients’ ovarian response status, there are a number of available ovarian stimulation protocols offered by IVF clinics. The protocols generally comprise of administering a combination of gonadotrophin analogs namely gonadotropins-releasing hormone (GnRH) agonist or antagonist, estradiol inhibitors, recombinant follicle-stimulating hormone (FSH), and luteinizing hormone (LH) (Gallos et al., 2017). While the drug regimens hold paramount importance for IVF and have been established and refined through years of clinical practice and research, the incidence of asynchronous ovarian response persists (Braga et al., 2020). This is evident in the miscellaneous yield of abnormal, immature, and poor-quality oocytes at retrieval. Furthermore, a portion of the immature oocytes would not progress to maturity during *in vitro* culture and could not be subjected to sperm injections while the poor-quality oocytes are at risk of forming aneuploid embryos (Figueira et al., 2010; Bosch et al., 2016). Hence, oocyte quality assessment is carried out prior to syngamy as a predictive marker of oocyte competence and IVF outcomes.

To date, the oocytes quality assessment is mostly based on morphology (Wang and Sun, 2007). The morphological grading system is mainly based on extra-cytoplasmic and cytoplasmic observations, which may give insight into the nuclear and cytoplasmic maturity state (Balaban et al., 2012). A spectrum of publications has supported the relevancy of one or more of the oocyte morphological characteristics in predicting the quality of the subsequent stages of embryo development. Tilia et al. (2020) inferred that oocyte meiotic spindle morphology is correlated to embryo developmental stages and embryo ploidy and that the oocyte morphology scoring system could be useful in determining embryos competent to achieve successful pregnancy (Lazzaroni-Tealdi et al., 2015). However, the accuracy of this morphological grading method in predicting IVF outcomes is still unconvincing due to its subjectivity and the lack of correlation to the quality or competence of the oocytes (Ruvolo et al., 2013). This has led to the current trend of research which attempts to utilize more definitive molecular parameters, such as biomarkers, in outlining the predictive models for IVF outcomes.

A few studies on the transcriptomes of COC have become prominent over the years due to the ease of deriving cumulus cells without jeopardizing the conditions of the oocytes (Wyse et al., 2020). Through methodologies for genotyping and gene expression analysis mainly DNA microarray, RT-qPCR (Reverse-Transcription quantitative Polymerase Chain Reaction), and Next Generation Sequencing (NGS), a large number of vital biomarkers and their roles were unveiled. Correspondingly, complex signal transduction pathways involved in oocyte maturation were gradually assembled, heightening our understanding of the pathophysiology of immature oocytes obtained during a controlled ovarian stimulation in IVF (Chronowska, 2014). The implementation of COC biomarker analysis as an effective IVF predictor could ultimately safeguard patients’ assurance throughout the program as they prepare for the next step. These biomarkers were not only anticipated to revolutionize oocyte selection in IVF but also pave attainable approaches that could improve *in vitro* maturation of oocytes, thus possibly increasing the success rate of IVF.

**FIGURE 1:**
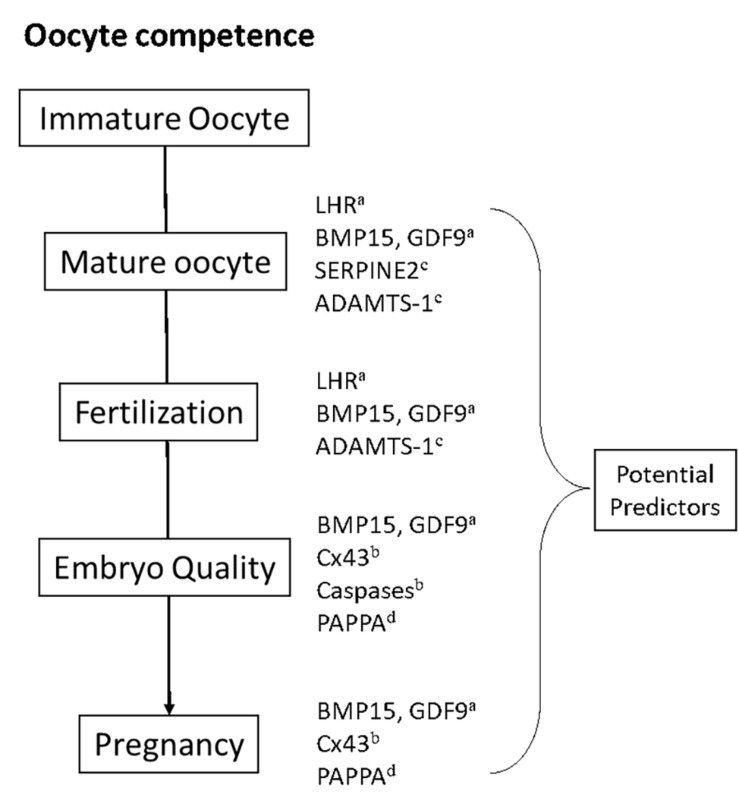
Oocyte competence in IVF could be defined as the ability of an oocyte to achieve the mature oocyte stage, fertilization, embryo stage, and ultimately pregnancy. Expression of the COC-derived genes discussed in this study have exhibited significant predictive values toward the respective IVF stages. ^*a,b,c,d*^ refers to the occyte maturation process that the gene regulate: ^*a*^Nuclear maturation, ^*b*^Apoptosis, ^*c*^Extracellular matrix remodeling, ^*d*^Steroid metabolism.

Findings on a total of eight biomarkers were dissected in this study (Table 1). These biomarkers are mainly regulatory RNA, proteins, receptors, growth factors, gap junctions, and proteases that are involved in the complex process of oocyte maturation. Most prospective studies are selected with subjects of discussion being the evidence-based breakthroughs of these biomarkers as possible prognostic indicators for an IVF cycle (Figure 1). Notably, some biomarkers have different or interconnected modes of action in regulating oocyte maturation and are hereby assorted based on the processes that they associate with during oocyte maturation.

**TABLE 1:** Summary table of eight cumulus biomarkers and their significance in assessing oocyte development competency and/or IVF outcomes.

Authors	Biomarkers	Oocyte maturation event	Detection method	Sample size	Key findings
Maman et al. (2012)	LHR	Nuclear maturation	RT-PCR	Granulosa cells from 70 IVF patients and 20 IVM (*in vitro* Maturation) patients	Expression of LHR is higher in metaphase II oocytes than in metaphase I and germinal vesicle oocytes (*P* < 0.05), but overexpression is associated with low fertilization capacity (*P* < 0.05)
Wiweko et al. (2019)	LHR		RT-PCR	Granulosa cells from 10 poor responder IVF patients and 20 non-poor responder patients	In poor responders: Positive correlation of LHR expression with oocyte maturity (*r* = 0.267), oocyte morphology (*r* = 0.267), and fertilization rate (*r* = 0.430) In non-poor responders: Negative correlation of LHR expression with oocyte maturity (*r* = −0.552), morphology (*r* = −0.164), and fertilization rate (*r* = −0.340)
Gode et al. (2011)	BMP15, GDF9		Western blot	81 individually derived cumulus-oocyte complexes (COCs) and follicular fluids collected from the first retrieved follicle of 81 IVF/ICSI patients	Nuclear maturation of oocytes (*P* < 0.05) and quality of embryos (*P* < 0.05) are significantly correlated with the level of mature GDF9 in follicular fluid
Li et al. (2014)	BMP15, GDF9		qPCR.	2,426 COCs from 196 IVF/ICSI patients	Expression of GDF9 and BMP15 mRNAs in cumulus granulosa cells were highly correlated with oocyte maturation, fertilization, and embryo quality (*P* < 0.05). Pregnancy predicting capacity is evident (GDF9 with 4.82 cut-off value, 82% sensitivity, and 64% capacity; BMP15 with 2.60 cut-off value, 78% sensitivity, and 52% capacity)
Hasegawa et al. (2007)	Cx43	Apoptosis	RT-PCR	105 CC samples from 29 IVF/ICSI patients	No relationship of Cx43 with fertilization and cleavage rate. Cx43 expression was significantly lower in good morphology cleavage-stage embryos compared to other groups (*P* = 0.035)
Wang et al. (2009)	Cx43		RT-PCR, Immunofluorescence microscopy, Western blot, gap junctional coupling assay	Cumulus cell (CC) samples from 115 patients	A positive correlation (*P* < 0.05) of Cx43 level with embryo quality (cleavage rate and morphology). Cx43 level is significantly higher in patients who became pregnant (*P* < 0.01)
Salehi et al. (2017)	Pro apoptosis proteins (Caspase-3, Caspase-7)		RT-PCR	100 CC from 20 PCOS IVF patients and 100 cumulus cells from 20 normal IVF patients (control group)	Caspase-3 and Caspase-7 expression were higher in PCOS patients. Significant correlation of embryo quality with Caspase-3 (*P* = 0.0016) and Caspase-7 (*P* = 0.084) expression in PCOS patients
Devjak et al. (2012)	SERPINE2	Extracellular matrix remodeling	Microarray, Quantitative real-time PCR (qPCR)	46 CC samples from 21 patients	Positive correlation between microarray and qPCR data (*r* = 0.98, *P* = 0.02) indicating lower expression of SERPINE2 mRNA in cumulus cells of mature oocytes than in immature oocytes
Li et al. (2015)	SERPINE2		RT-PCR	308 COC samples from 40 IVF patients (53 immature oocytes and 255 mature oocytes)	Expression of SERPINE2 was significantly lower in cumulus cells of mature oocytes than in immature oocytes (*P* = 0.0002)
Yung et al. (2010)	ADAMTS-1		Quantitative real-time PCR (qRT-PCR), Western blot	CC from 25 patients	ADAMTS-1 is associated with larger and more mature follicles and is positively correlated with oocyte fertilization capacity
Kordus et al. (2019)	PAPPA	Steroid metabolism	RT-PCR	163 CC samples from 15 patients	PAPPA expression level is significantly higher in CC of oocytes that led to euploid embryos resulting in a live birth (*P* < 0.05, *OR* = 4.591)
Wyse et al. (2020)	42 potential oocyte maturation biomarkers	Transcriptomics of cumulus cells	RNA sequencing, differential expression, pathway analysis, Quantitative real-time PCR (qPCR)	22 COCs from 11 patients	Differentially expressed genes identified between oocytes of different maturity

## Oocyte Nuclear Maturation

Nuclear maturation that is characterized by germinal vesicle breakdown denotes the continuation of meiosis I division from prophase I to metaphase II. The majority of intra-ovarian oocytes are arrested at prophase I due to the low activity of maturation promoting factors (MFP), which consist of two sub-unit proteins namely cyclin-dependent kinase 1 (CDK1) and Cyclin B1. Physiologically, low activity of intra-oocyte MPF is instigated by the presence of adenosine monophosphate (cAMP) produced by intra-follicular somatic cells or the oocytes itself (Edry et al., 2006), and cyclic guanosine monophosphate (cGMP) produced by follicular somatic cells (Norris et al., 2009). Both of these molecules are transported into the oocyte via gap junctions. cAMP prevents the activation of CDK1 through the protein kinase A (PKA) signaling pathway, while a high concentration of cGMP inhibits the degradation of cAMP by phosphodiesterase PDE3A enzyme. The inhibitory actions of the signaling molecules altogether ensue the low MPF activity that in turn leads to maturation arrest (Adhikari and Liu, 2014). Luteinizing hormone (LH) plays a significant role in counteracting the inhibitory signaling molecules by restricting the translation of Cx-43 protein, which is an essential backbone protein for the construction of gap-junction (Kalma et al., 2004). Diminished expression of Cx-43 protein alters cell-to-cell communication between intrafollicular somatic cells leading to the low levels of intra-oocyte cAMP and cGMP; thus, inducing the continuation of meiosis prophase I up till the metaphase II arrest. At this stage, oocyte nuclear maturity is discernible by the presence of the first polar body and the emergence of a meiotic spindle that is otherwise not observed in immature oocytes.

LH is engaged in regulating the expression of specific genes that concern the EGF (Epidermal Growth Network) and gap junction permeability to achieve nuclear maturation and finally oocyte competency prior to fertilization (Conti et al., 2012). LH receptor (LHR) plays a central role in the stimulating mechanism of the hormone and the availability of the G-protein coupled LH receptors in human granulosa cells make it a valuable biomarker for oocytes nuclear maturation. Findings by Maman et al. (2012) describe that LHR mRNA is expressed higher in granulosa cells of mature oocytes than granulosa cells of immature oocytes, metaphase 1, and germinal vesicle oocytes, but higher expression of LHR mRNA was found to be associated with decreased fertilization rate. Moreover, Wiweko et al. (2019) investigated the expression of LHR mRNA on oocyte maturation and fertilization rate of two groups of IVF patients, namely poor responders, and non-poor responders. The results indicated that the LHR mRNA relative value was higher but not significant in poor responders compared to non-poor responders. Furthermore, the expression of LHR mRNA had a positive correlation with oocyte maturity and fertilization rate in the poor responders, while a negative correlation was observed in the non-poor responders. Nonetheless, the studies have implied that the expression of LHR is indeed associated with promoting oocyte nuclear maturation. However, there are some limitations in utilizing LHR as biomarkers. Overexpression of LHR has been presumed to give adverse impacts such as low fertilization rate hence expression thresholds of LHR that signify pro-maturation need to be determined. Moreover, as stated by Jeppesen et al. (2012), LHR protein expression might be too low in the granulosa cells for proper detection through RT-qPCR resulting in a tentative conclusion. There are some other notable findings that aim to evaluate prospective biomarkers and could be the alternative or complementary biomarkers. A G protein-coupled receptor, GPR3, that was mainly described and assessed in rodents was associated with meiotic arrest in human oocytes (DiLuigi et al., 2008).

Known as oocyte secreted factors (OSF) that belong to the transforming growth factor-β (TGF-β) family, growth differentiation factor-9 (GDF-9), and bone morphogenetic protein 15 (BMP-15), are proposed to have a potential value as non-invasive markers for determining oocyte developmental potential. In the ovary, GDF-9 and BMP-15 play pivotal roles during folliculogenesis, oocyte maturation, and ovulation (Gilchrist et al., 2004). Although OSFs are mainly secreted by the oocytes, granulosa cells are also known to express the GDF-9 and BMP-15. During the initial discovery, it was believed that GDF-9 and BMP-15 exist as homodimers formed through non-covalent linkage. However, a recent study has proven that both proteins could prevail as heterodimers (GDF-9/BMP-15), later known as cumulin, which possess a higher capability in inducing the proliferation of granulosa cells compared to the respective homodimer forms (Mottershead et al., 2015).

The clinical value of GDF-9 in the follicular fluid has been shown in a study conducted by Gode et al. (2011), in which mature forms of GDF-9 protein were significant in association with oocyte nuclear maturation. Similarly, a study that involved 196 infertile couples undergoing IVF, revealed that the COCs of women who achieved pregnancy expressed higher GDF-9 and BMP-15 mRNA concentrations than those who did not achieve pregnancy (Li et al., 2014). Furthermore, the study has also successfully formulated the cut-off values of both markers (4.82 and 2.60, respectively, for GDF-9 and BMP-15) for predicting pregnancy with sufficient sensitivity (78and 82%, respectively, for GDF-9 and BMP-15). Both studies have provided essential information on the expression of GDF-9 and BMP-15 in infertile couples undergoing IVF and the promising clinical use of the OSF as non-invasive markers to predict oocyte competence.

## Apoptosis in Oocyte Maturation

Apoptosis or programmed cell death has been known to play a role in influencing the oocyte maturation process. Apoptotic events in granulosa cells cause interference of the granulosa cells-oocytes communication, limiting the maturation-inducing factors (hormones, proteins, and metabolites) supply that are necessary for oocytes to reach meiotic competency (Chaube et al., 2014). Granulosa cells that do not have the access to all the necessary metabolites would gradually promote the autophagy mechanism before eventually inducing apoptosis mechanism (Altman and Rathmell, 2012). Gap junctions that act as channels for these necessary metabolites play an important role in regulating cell death, survival, and nutrients uptake.

Gap junctions, which allow cell-to-cell communication is found among intrafollicular cells, i.e., mural granulosa cells, cumulus granulosa cells, and oocytes (Anderson and Albertini, 1976; Gilula et al., 1978). Connexins are essential in forming intercellular membrane channels of gap junctions. Connexin (Cx43), particularly, is expressed in granulosa cells and is associated with cell death and survival, and oocyte maturation in model animals (Vozzi et al., 2001; Krysko et al., 2004), making it a potential biomarker for human oocyte quality.

Wang et al. (2009) found that Cx43 expression level had a positive correlation to embryo quality as in morphology and pregnancy rate. It was presumed that low expression of Cx43 is also followed by low availability of fundamental nutrients, creating a metabolic shortage in oocytes that would impact the oocytes in the later developmental stages. This discovery is contradictory to findings by Hasegawa et al. (2007), whereby Cx43 expression was found to be lower in the good-morphology embryo group compared to other groups. This evidence is supported by the fact that LH surge, which is central in meiotic resumption inhibits the translation of gap junction proteins (Kalma et al., 2004). The inhibition of gap junction protein is presumably to limit the transfer of meiotic maturation arresting factor, cAMP and cGMP, from the cumulus cells to oocytes (Norris et al., 2008). In summary, the limitation of Cx43 as a biomarker prevails in the uncertainty as to whether the decreased or increased expression of Cx43 is related to favorable oocyte quality.

Caspase is one of the other pro-apoptotic factors expressed in granulosa cells that could become potential biomarkers. Caspase, a cysteine protease, causes the inactivation of components for physiological processes and morphological changes during apoptosis. Caspase-3 and caspase-7 expressions were found to be higher especially in polycystic ovary syndrome (PCOS) and have a negative correlation to embryo quality (Salehi et al., 2017). Evidently, caspase-3 is the main factor in apoptosis through its association with chromatin condensation, DNA fragmentation, nuclear breakdown, and plasma membrane protrusion, while caspase-7 mainly contributes to the reactive oxygen species (ROS) accumulation and detachment of cells from the extracellular matrix (ECM) (Brentnall et al., 2013). FOXO3 is another gene that is highly expressed during apoptosis and correlated positively with poor quality oocytes in PCOS patients (Mikaeili et al., 2016).

## Oocyte Extracellular Matrix Remodeling

The integrity of the COC extracellular matrix is vital for the bidirectional intercellular communication between oocytes and their surrounding cumulus cells. During oocyte maturation, the dynamic surge of gonadotropins and oocyte-secreted paracrine factors stimulate several genes that participate in a process known as cumulus expansion. The expressed proteins acquire different functions including gap junctions and proteases which predominantly regulate the remodeling of tissues during folliculogenesis; all acting within the intercellular spaces of cumulus cells to promote oocyte maturation (Robker et al., 2018).

*SERPINE2* encodes for the serine protease inhibitor E2 that is not only indirectly associated with tissue plasminogen activator in degrading matrix proteins but also plays a role as sperm decapacitation factor. Li et al. (2015) highlighted the considerable difference in *SERPINE2* gene expression between immature and mature oocytes in which RT-qPCR of RNA extracted from 308 COCs revealed a significantly lower expression of *SERPINE2* mRNA in a cumulus of mature oocytes compared to the immature cohort (*p* = 0.0002). This corroborated a previous study that indicated *SERPINE2* as one of the 116 genes to be differentially expressed in the DNA microarray analysis of cumulus samples between mature and immature oocytes (*p* = 0.02) (Devjak et al., 2012). Furthermore, the study particularly disclosed no substantial difference in cumulus cell gene expression with regard to the ovarian stimulation protocols. Recent experimentation which managed to construct an NGS library to discern the transcriptomics of cumulus cells also displayed a fourfold decrease in *SERPINE2* expression in the immature oocyte cohort (Wyse et al., 2020). Concurrently, Wyse et al. (2020) also identified a higher expression of cumulus expansion-associated ADAM-metallopeptidase with thrombospondin type 1 motifs-1 (*ADAMTS-1)* in COC of mature oocytes. This was previously exhibited in a study that specified its essential role in the follicular rupture process. *ADAMTS-1* expression was found to be correlated with oocyte fertilization capacity in which oocytes that underwent successful fertilization acquired a threefold higher expression of cumulus *ADAMTS-1* compared to those that failed to fertilize (Yung et al., 2010).

While these studies might have proven the competency of *SERPINE2* and *ADAMTS-1* as biomarkers for oocyte competency, they are limited to the small immature oocyte sample sizes within the same cycles (controlled ovarian stimulation in IVF typically produces more mature oocytes) hence a larger prospective study is still mandatory. A study on bovine follicles demonstrated the influence of gonadotropins on *SERPINE2* expression in which FSH was found to manifest in the increased *SERPINE2* expression while LH surge before ovulation decreased its expression (Bédard et al., 2003). However, further research is still required to unveil the pathology that leads to the turnarounds in the expression of *SERPINE2* and *ADAMTS−1* in the cumulus of immature oocytes so that the appropriate intervention could be formulated to improve *in vitro* maturation.

## Oocyte Steroid Metabolism

Steroidogenesis in animal models has been shown to directly impact the meiosis of oocytes and contribute to spontaneous mammalian oocyte maturation (Jamnongjit and Hammes, 2005). While their exact regulatory mechanisms are still rather bleak, steroids secreted by theca and granulosa cells in response to growth factors have been established to act as secondary messengers in pathways that lead to cumulus expansion and thus oocyte maturation. Of utmost interest, studies that explore the metabolomics of cumulus cells have unveiled pappalysin-1 (*PAPPA*) mRNA as a potential predictor of oocyte maturation status and, to a certain degree, the euploidy status of embryos and pregnancy outcomes in IVF (Kordus et al., 2019). *PAPPA* is responsible for the expression of metalloproteinases which actively cleaves insulin-like growth factor binding proteins (IGFB4) to provide the supply of intrafollicular IGF peptides that in turn function as co-gonadotropin for the stimulation of steroidogenesis. Kordus et al. (2019) noted higher *PAPPA* expression levels in cumulus cells of mature oocytes that ultimately developed to become euploid embryos compared to the group of immature oocytes that culminated in arrested embryos. Findings of the study also suggested a computable interplay between the expression of *PAPPA* and other oocyte maturation-associated mRNAs such as *AREG* (amphiregulin) and *LHCGR* (luteinizing hormone/choriogonadotropin receptor) which is a valuable steppingstone toward constructing an ideal predictive model that could gauge the prognosis of IVF.

Another steroidogenesis-associated gene that is also adversely expressed differentially between mature and immature oocytes is *HSD17B1* which encodes Hydroxysteroid (17beta) dehydrogenase 1 (Järvensivu et al., 2015). The enzyme, in the presence of cofactors, regulates the reversible catalytic reaction of converting estrone to estradiol which possesses a higher estrogenic activity. *HSD17B1* has been extensively evaluated in animal models and the recent transcriptomic analysis of human COC samples corroborates the significantly reduced expression of *HSD17B1* in mature oocytes (Wyse et al., 2020), commencing possible research in the mechanism and roles of the gene in promoting the maturation process. Likewise, further research worth investigating would be on the affiliation of *PAPPA*, and *HSD17B1* mRNA expression level in mature oocytes with their respective pregnancy outcomes. Such studies, however, encounter inevitable challenges that demand a large sample of single embryo transfer procedures while still minimizing the clinical and physiological heterogeneity among the samples.

## Discussion

Oocyte maturation is a complex process consisting of inter-and intra-connected signaling pathways that are regulated by a series of genes traceable within the COC. Gene expression analyses of RNA samples extracted from COC have revealed that certain genes were particularly expressed differently in oocytes of different maturity hence extending their potential purpose as biomarkers capable of predicting IVF outcomes. The most promising biomarkers thus far are GDF-9 and BMP-15 with proven sensitivity and specificity of predicting pregnancy in a study with the largest sample size among others included here. Furthermore, addition of these growth factors has shown to increase the efficiency of *in vitro* maturation, suggesting their significant roles in promoting oocyte maturation. The potency of other biomarkers such as SERPINE2 and Cx43 require additional investigations due to the relatively small sample size and contradicting results with previously established findings. Meanwhile, recognizing confounding variables are imperative to identify underlying conditions in IVF patients which may affect the expression of these biomarkers such as the significantly higher expression of Caspase proteins in PCOS patients. Although substantial *in vitro* evidence was presented to justify the potential functions of these biomarkers, the clinical practice of examining non-invasive oocyte biomarkers as IVF prognostic measures is still far from achievable. Ideally, such approaches would demand the acquisition of transcriptomic profiles of every oocyte retrieved during ovum pick up, enumerating the expression levels of all the genes mentioned above, in addition to many more.

A large number of COC biomarkers are regulated by FSH and/or LH stimulation. Poor response toward the gonadotrophins have been shown to downregulate genes which, in turn, are associated with the increased p53-pathway for apoptosis, resulting in a restricted maturation process, as observed in immature COCs (Wyse et al., 2020). Contrarily, adequate gonadotrophinresponses characterized by the upregulation of genes for the biosynthesis of estrogen and androgen are displayed in mature oocytes (Wyse et al., 2020). While these findings have collectively added knowledge to the understanding of oocyte maturation process, correlating the expression level of these genes with the tracking of individual oocyte outcome throughout the IVF program is required before the genes could be denoted as potential IVF predictors. In that note, in the transcriptomic profiling of COCs, there are several novel discoveries on the differential expression of other explored genes between the cohorts of mature and immature oocytes such as genes encoding for Epiregulin (EREG) in cumulus expansion and Phosphodiesterase 3A (PDE3A) in cytoplasmic maturation (Wyse et al., 2020). Nonetheless, the exact mechanism and significance of these genes as indicators for assessing oocyte competence and thus the IVF outcomes are still rather bleak.

While plenty of other biomarkers were not discussed here, this review provides insights on the current progress of studies pertaining to the evaluation of cumulus biomarkers. Common challenges such as the difficulty in obtaining homogeneous study subjects were highlighted, including the imbalance number of mature and immature oocytes retrieved during ovum pick-up, which understandably led to the resultant small sample sizes. Additionally, in the attempts to correlate these biomarkers with IVF outcomes, a large assortment of confounding variables and appropriate statistical methodologies need to be recognized to provide convincing results and evade contradictory findings. It is also imperative to expedite the identification of factors that culminate to the shift in gene expression levels between mature and immature oocytes, bringing about another limitation specifically in the selection of the appropriate model organisms. Other routes of research worth investigating would be to classify metabolite analogs affiliated with the biomarkers and evaluate their effects during *in vitro* maturation process, with hopes for the possibility to boost the competency of oocytes within laboratory settings.

## Conclusion

In conclusion, the roles of eight cumulus biomarkers in promoting oocyte maturation and their expression profiles in oocytes of different maturity have been well-established to possess statistical values in predicting an IVF cycle. However, the feasibility of assessing these biomarkers as an add-on prognostic procedure in IVF is still restricted due to sampling challenges. Indispensable investigations on the disputable molecular mechanisms are also still necessary for the realization of IVF predictive models that are based on the above-mentioned biomarkers.

## Author Contributions

BS drafted the early version of this manuscript. BW, AAJ, DI, and RM critically reviewed and revised the content. All authors contributed to the article and approved the submitted version.

## Conflict of Interest

The authors declare that the research was conducted in the absence of any commercial or financial relationships that could be construed as a potential conflict of interest.

## Publisher’s Note

All claims expressed in this article are solely those of the authors and do not necessarily represent those of their affiliated organizations, or those of the publisher, the editors and the reviewers. Any product that may be evaluated in this article, or claim that may be made by its manufacturer, is not guaranteed or endorsed by the publisher.
